# Transoral thyroid surgery vestibular approach

**DOI:** 10.1515/iss-2021-0033

**Published:** 2022-06-17

**Authors:** Elias Karakas, Günther Klein, Stefan Schopf

**Affiliations:** Dept. of General-, Abdominal- and Endocrine Surgery, Hospital Maria Hilf, Alexianer GmbH, Krefeld, Germany; Dept. of General Surgery, Landesklinikum Wiener Neustadt, Wiener Neustadt, Austria; Dept. of General-, Abdominal- and Endocrine Surgery, RoMed Hospital, Bad Aibling, Germany

**Keywords:** remote access, thyroid, transoral

## Abstract

**Objectives:**

Transoral thyroid surgery vestibular approach (TOETVA) is a novel and feasible surgical technique that allows for cervical surgery without visible incisions. TOETVA represents a new frontier in endocrine surgery since aesthetic results play a more and more decisive role in elective surgery. However, acceptance is different around the world with widespread prevalence in Asian countries and some high-volume centres in the US. While inclusion criteria for TOETVA are limited regarding size and volume a combination with other extracervical techniques like the retroauricular endoscopic cephalic access thyroid surgery (EndoCATS) approach or transaxillary access is an option.

**Methods:**

TOETVA is carried out through a three-port technique placed at the oral vestibule. Originally one 10-mm port for a 30° endoscope and two additional 5-mm ports for dissecting and coagulating instruments are used. Alternatively, one 5-mm and one or two 3 mm ports can be used. CO2 insufflation pressure is set at 6 mmHg. An additional device to optimize gas outflow for optimum view might be helpful. An anterior cervical subplatysmal space is created by hydrodissection from the oral vestibule to the sternal notch, laterally to the sternocleidomastoid muscle. Conventional endoscopic instruments are used. Combination of TOETVA with a modified retroauricular access includes insertion of a 10–12 mm trocar placed subcutaneously via a skin incision on the scalp, behind the ear by blunt dissection.

**Results:**

Since Anuwong published the first case series of 60 patients who underwent scarless thyroidectomy via the lower vestibule of the mouth with excellent results in 2016 almost 1,000 cases are reported in literature to date with comparable results especially regarding traditional complications. In contrast to other extracervical approaches, areolar or axillary for example, the transoral access route is short and the dissection planes are rather like transcervical surgery. Surgical indications and contraindications have been modified since its first description and are partly institution specific to date. To amend indications combination with other extracervical techniques is an option. In addition, patients must carefully be selected for and surgeons` candidacy is of utmost importance in transoral surgery.

**Conclusions:**

Transoral surgery will likely continue to gain attraction as surgeons become more experienced with the technique. With increased operative use and surgeon experience the gap in conventional outcomes between transoral surgery and the transcervical approach will narrow, with both operative time and the incidence of specific complications diminishing. Experience in thyroid and endoscopic surgery is required to achieve excellent results with low complication rates. However, the new transoral technique is related to novel complications that must be evaluated.

## Introduction

Since the 1990s various techniques like transcervical video assisted surgery, and extracervical transaxillary, breast or postauricular (EndoCATS) procedures have been developed in thyroid and parathyroid surgery with the aim to minimize or completely avoid a visible scar in the neck. Prior to this thyroid and parathyroid surgery has been safely performed via an anterior neck incision for more than 100 years. First step to optimize cosmetic results and health related quality of life (HRQL) was to relocate cutaneous incisions to less suspicious locations like the areolar or axillary incisions, which can effectively minimize the cosmetic burden in some patients [1, 2].

Development of new techniques was triggered by technical progress on the one hand and patients burden of suffering from unsightly scars and patients desire to avoid such scars in the neck on the other hand. Notably, about 20% of patients will experience feelings of self-consciousness years after surgery and more than 10% will consider further treatments as far as plastic surgery to improve the scar in the neck [3, 4]. Despite the improved of local cosmesis, other extracervical techniques are challenging due to unfamiliar dissection planes, longer routes to the central neck leading to steep learning curves for surgeons with long operative times and novel complications. Consequently, the adoption of these alternative techniques was slow, especially in Western countries [5].

Finally, the emergence of natural orifice transluminal endoscopic surgery (NOTES) led to the development of transoral endoscopic thyroid and parathyroid surgery. Transoral endoscopic surgery has been carefully investigated since 2008 initially performed throughout a sublingual approach representing the only surgical technique for thyroid and parathyroid surgery that does not have any cutaneous incision. However, the sublingual approach, originally performed in Germany, was discontinued after substantial experimental and few clinical studies because of significant drawbacks and disadvantages like restrictive surgical space and view, inappropriate instrumentation, transient but severe postoperative swelling, swallowing problems and pain [6].

In 2016, Anuwong published the first case series of 60 patients who underwent scarless thyroid surgery via the lower vestibule of the mouth with excellent outcomes [7]. Currently Anuwongs` personal experience exceeds 2000 cases (unpublished data) and multiple institutions around the world have adopted this new technique with similar good results in almost 1,000 cases, attracting patients who are interested in avoiding a neck scar [7], [8], [9], [10], [11], [12], [13], [14].

The authors aim is to update the reader on the status of transoral thyroid and parathyroid surgery via the vestibular approach regarding technique and technical modifications, indications, advantages and potential new problems.

## Transoral surgery vestibular approach – qualification and requirements

Surgeon skills and qualification including experience in endoscopic surgery are of utmost importance for procedure safety. Proficiency in the standard procedures of central neck surgery, including thyroidectomy, parathyroidectomy and central neck dissection in case of necessary conversion to open surgery is prerequisite [15, 16]. An adequate volume is required to overcome the learning curve. The authors recommend participation in hands on cadaver training ideally accompanied by proctoring by surgeons experienced in transoral surgery within the first cases. Subsequently an increase in procedural proficiency might be observed after 11 cases [9, 17]. However, some authors assume that more than 50 cases are necessary to overcome the learning curve [18].

## Transoral surgery vestibular approach – technique and modifications

**Figure 1:**
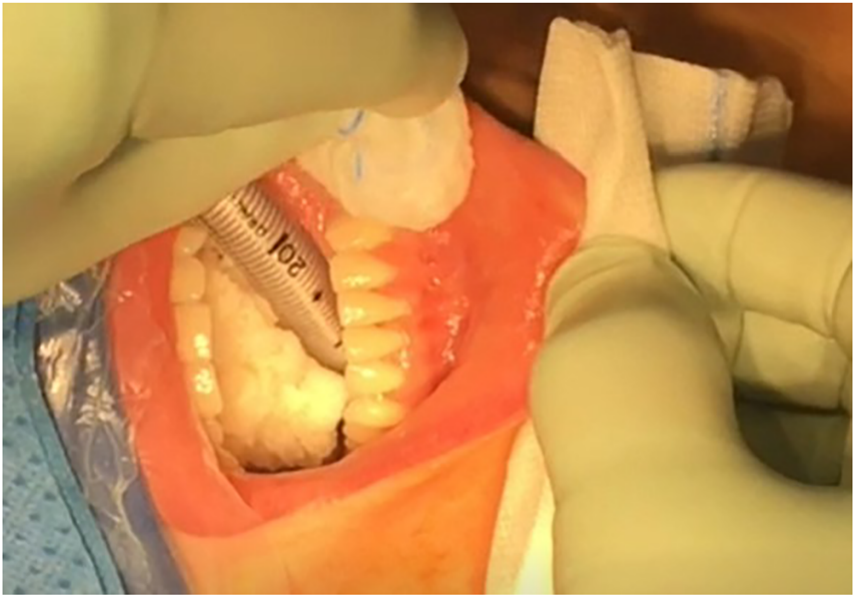
Transoral intubation and mucosal disinfection.

**Figure 2:**
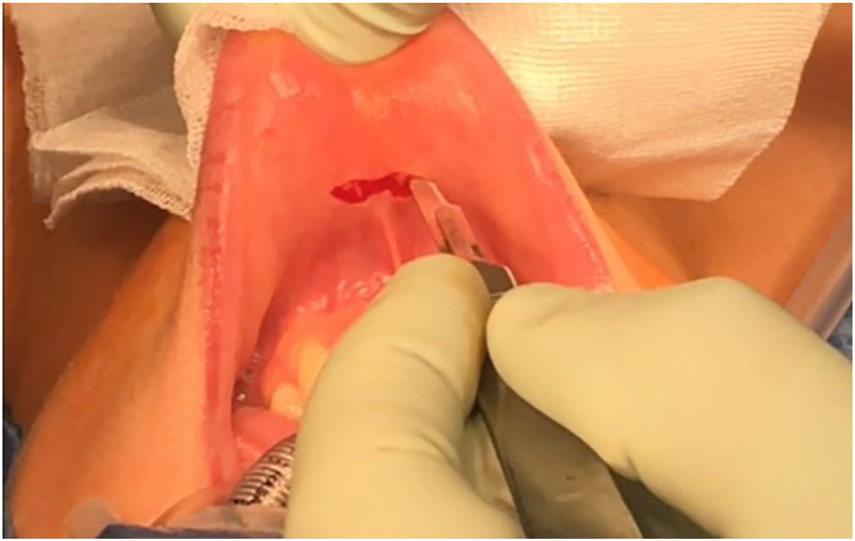
“Cold” mucosal incision to avoid thermal damage.

**Figure 3:**
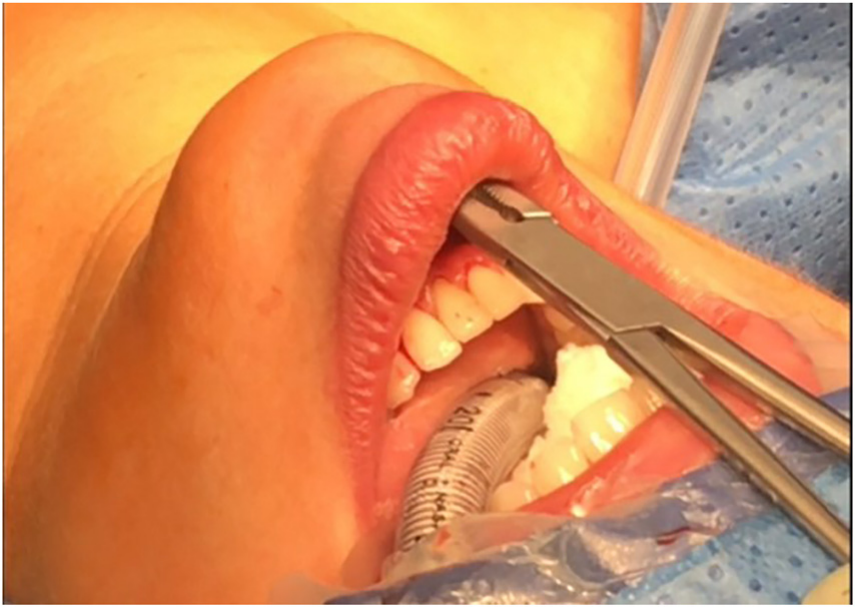
Blunt dissection to the thyroid region to avoid thermal skin damage.

Three incisions are made in the lower vestibule of the mouth. Prior to this the oral cavity should be cleansed with chlorhexidine solution (Figure 1). The midline incision is made just above the inferior labial fraenulum with a “cold” scalpel to avoid thermal injury (Figures 2 and 3). The other two 5 mm incisions are made just medial to the lip vermillion border near the labial commissure. These two incisions are made lateral/posterior from the canine teeth as far away from the branches of the mental nerves as possible, which have been attributed to less mental nerve injury and tearing of the lip commissures [9, 16, 18].

To reduce tissue trauma several modifications are possible and recommended:

A 5 mm instead of a 10–12 mm trocar combined with a 5 mm 30° camera can be used in the midline. Use of the midline 5 mm trocar may facilitate initial trocar placement. It is minimal invasive and space saving during surgery and not confined to particular cases. To extract taller specimen, tissue in the vestibular and chin area can be stretched at the end of surgery if necessary. Otherwise, additional extracervical or a submental approach can be used to extract huge thyroid specimen. 3 mm trocars and instruments are available and applicable instead of the lateral 5 mm incisions to reduce invasiveness.

**Figure 4:**
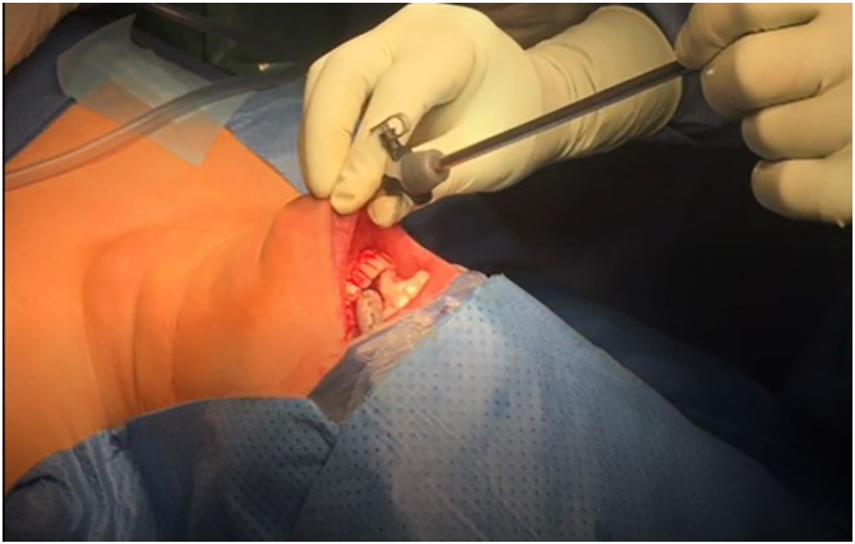
Trocar placement 5 mm instead of 12 mm in the midline.

**Figure 5:**
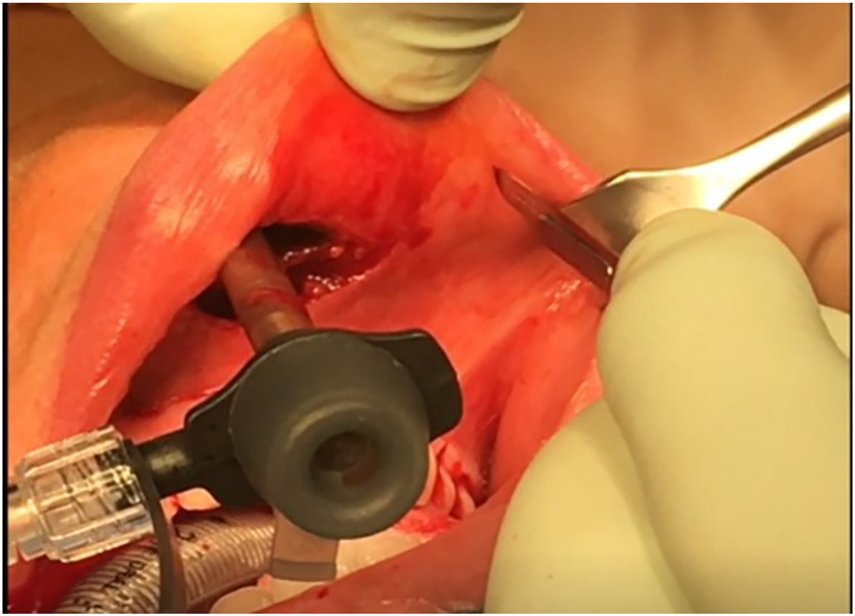
Lateral incision close to the lip vermillion to avoid mental nerve injury.

To widen and create the midline access blunt instead of thermal dissection is recommended to avoid the risk of lip or skin burn in the chin region (Figures 4 and 5).

Like conventional surgery all procedures must be performed using intraoperative nerve monitoring (IONM). An endotracheal tube with an electrode for IONM is placed transorally (Figures 1 and 2). The authors recommend a long reusable stimulating probe (e.g. Dr. Langer Medical, Waldkirch, Germany) applicable via one of the trocars. Alternatively, the stimulating probe can be inserted via an additional minimal (1 mm) skin incision in the neck [19].

**Figure 6:**
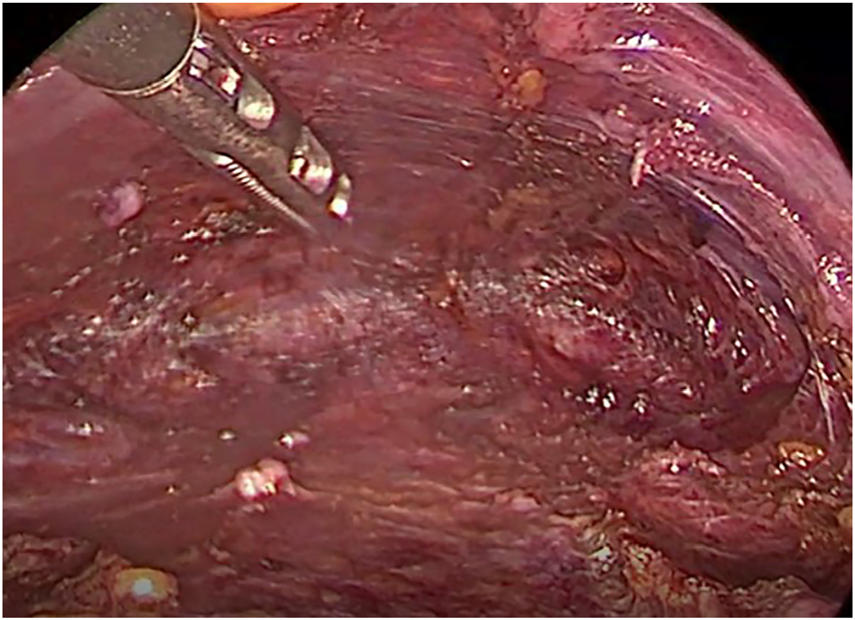
Subplatsymal space created by blunt and hydrodissection.

A subplatysmal space is created by hydrodissection using an epinephrine containing saline solution (1 mg/L) injected using a Verress needle. The subplatysmal space is widened using a blunt reusable dissector. High flow CO_2_ gas insufflation at a pressure of 6 mmHg is recommended and ventral and lateral retraction of the strap muscles using stiches placed from outside are used to maintain the subplatysmal working space from the thyroid cartilage to the sternal notch and to the medial border of both sternocleidomastoid muscles (Figure 6). An additional device to optimize gas outflow for optimum view might be helpful.

**Figure 7:**
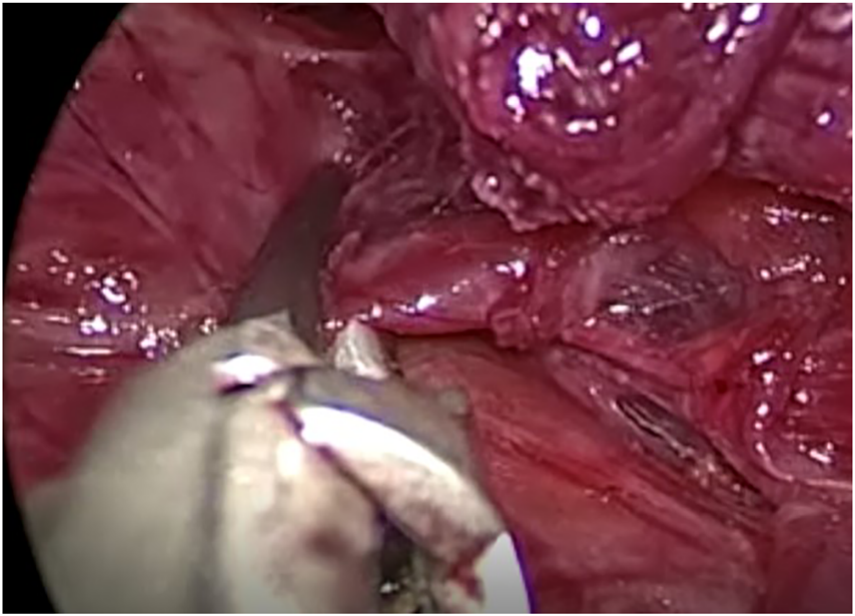
Division and dissection of the midline and the thyroid isthmus.

**Figure 8:**
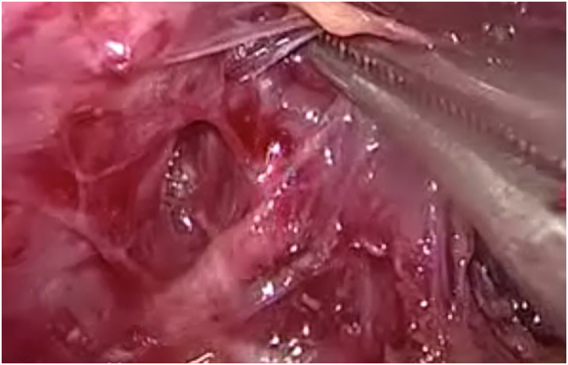
Lateral mobilisation and exposure of the recurrent laryngeal nerve.

Division of the strap muscles in the midline is followed by transection of the thyroid isthmus. After mobilization of the thyroid lobe the upper pole vessels are selectively sealed with a thermal device (Figure 7). Visualization and preservation of the parathyroid glands and the recurrent laryngeal nerve and stimulation of the vagal nerve is obligatory (Figure 8).

**Figure 9:**
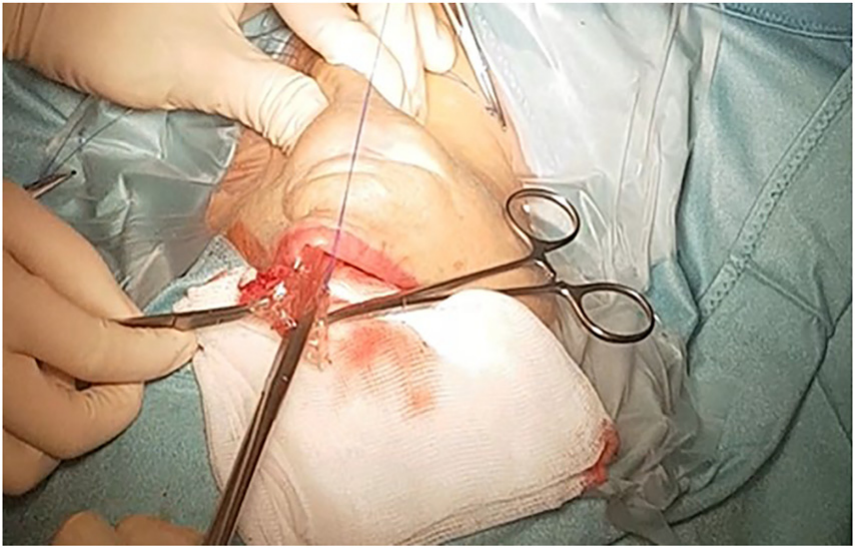
Transoral extraction of the thyroid specimen within an endobag.

**Figure 10:**
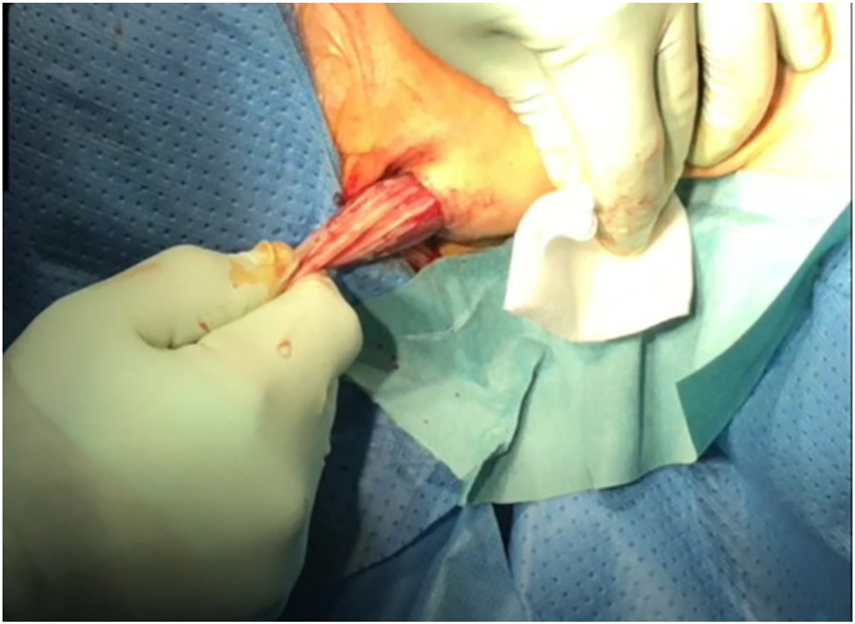
Retroauricular extraction of the thyroid specimen.

Removal of small thyroid or parathyroid specimen can be done through the midline incision within a retrieval bag (Figures 9 and 10). To extract huge specimen the authors combined the retroauricular, extracervical approach first described by Schardey and Schopf with TOETVA. The retroauricular approach was modified and simplified by subcutaneous blunt insertion of an additional 12 mm trocar instead of complex deep preparation in between the sternal and clavicular part of the sternocleidomastoid muscle using a special spatula [19].

Strap muscles can be adapted by transoral running suture at the end of the operation while the skin-platysm conjunction stays unaffected. Oral mucosa as well as retroauricular skin incision are closed using a 4/0 absorbable suture.

## Transoral surgery vestibular approach – indications and limitations

Primarily, patients must be motivated to avoid a skin incision, an anterior cervical scar or have a history of hypertrophic scarring. Up to 40 percent of patients undergoing thyroid or parathyroid surgery are eligible for transoral surgery considering current most widely accepted guidelines [8, 20, 21].

TOETVA/transoral thyroid surgery can be performed in both benign and malignant conditions. This and other surgical indications as well as contraindications have been changed since its first description in patients by Anuwong and are still practice or institution specific [9, 19, 21]. Regarding pure transoral surgery without an additional extracervical extraction access, indications are as follows:

Symptomatic grade I goitre and Grave’s disease can be approached through pure TOETVA. Thyroid gland size cut-offs include a diameter of 8–10 cm or estimated thyroid volume ≤45 mL, with a nodule size cut-off between 4 and 6 cm among benign or cytologically indeterminate (Bethesda II, III, IV) nodules. For patients with nodules that are suspicious for malignancy (Bethesda V) or confirmed well differentiated thyroid cancer without evidence of metastasis, a maximum nodule size of 2 cm has been utilized, which has been shown to be a safe threshold. If completion thyroidectomy is necessary, a repeat-TOETVA for the contralateral thyroid gland can be safely performed immediately after or within 3 weeks after initial surgery. Beyond that period, it may be appropriate to consider completion TOETVA after 6 months [8, 22], [23], [24], [25], [26]. Like in conventional surgery thyroiditis may aggravate preparation and mobilization of the thyroid and transoral surgery should be performed by well experienced surgeons.

Current TOETVA exclusion criteria include lateral neck or extrathyroidal disease extension, preoperative RLN paresis, prior transcervical neck surgery, oral abscesses/infection, or patient intolerance to general anaesthesia. Transoral surgery is not absolutely contraindicated after cervical neck surgery and neck radiation as well as patients age and body mass index (BMI) [8, 19], [20], [21], [22], [23], [24], [25], [26].

Combination of transoral surgery with extracervical approaches like the modified retroauricular EndoCATS access (TransOral Vestibular And Retroauricular Approach–TOVARA) allows for thyroid surgery without a visible scar in specimen with volume of more than 40 mL and may help to protect soft tissue, muscle, and nerve fibres in the vestibular and chin area during the extraction process especially in huge thyroid specimen [19, 27].

## Transoral surgery vestibular approach – advantages and options

(1) Main benefit of transoral surgery is the optimum cosmetic result. (2) With increasing experience and combination with other techniques inclusion criteria are broadening and exceeding other extracervical approaches. (3) The vestibular access is closer to the thyroid gland than that from the axilla, breast or retroauricular. In addition, surgical anatomical subplatysmal planes are respected. (4) Median central approach enables and facilitates bilateral cervical surgery including the central compartment. The angel of vision is cranial to caudal and more familiar for surgeons routinely involved in conventional thyroid surgery regarding the laryngeal and vagal nerves and parathyroids glands. (5) Surgery is done with conventional endoscopic or robotic instruments. (6) No adhesive tape is required postoperatively, and enoral mucosal healing is completed within 24 h. Complete body care and shaving is allowed immediately after surgery. Oral intake can be started in the evening after surgery and discharge from hospital is equal to conventional thyroid surgery.

## Transoral surgery vestibular approach – outcomes and potential complications

Own results regarding outcomes of 140 TOETVA, TOEPVA and TOVARA patients who have undergone isthmectomy, lobectomy, total thyroidectomy and parathyroidectomy are in line with results reported by other groups. Only one conversion was necessary in an obese young female (BMI 40) with a short neck and huge multinodular goitre (>150 mL) (0.7%) due to inadequate space.

In addition to common minor and major complications in thyroid surgery, like bleeding, vascular injury, RLN palsy, hypoparathyroidism or tracheal injury some procedure specific complications may occur in transoral surgery.

One female patient suffered from permanent RLN injury (1/204 nerves at risk, 0.5%) after surgery for Graves’ disease, and two patients had persistent mental nerve discomfort more than three months after surgery with only slight paraesthesia and clinical discomfort. Permanent hypoparathyroidism, CO_2_ embolism or emergency reoperation due to postoperative bleeding did not occur. Transient superficial haematoma of the skin region and neck may occur in some patients.

Transoral surgery to the neck is classified as ‘clean-contaminated’ and bears the risk of contamination and microbial allocation from the mouth into the thyroid region. In addition, extended operation time in transoral surgery may also contribute to higher risk of surgical site infections (SSI).

Considering preoperative intravenous antibiotic prophylaxis and oral mucosal disinfection the vestibular approach is not associated with an increased number of wound infections. Evaluation of white blood cell count (WBC) and C-reactive protein levels (CRP) in 75 patients with transoral thyroid surgery showed a transient WBC increase postoperatively, that returned to basic value within one week after surgery and without any clinical sign of infection. CRP levels were normal before and after surgery. However, in one patient oral antibiotic therapy was necessary due to transient erythema in the chin region which occurred 10 days after surgery and resolved completely without surgical intervention.

Another complication reported is skin flap burn in the chin region caused by thermal injury that occurred in one of our patients and fortunately, only a small 0.2 × 0.3 mm scar persisted. TOETVA patients had decreased postoperative pain compared with patients undergoing transcervical thyroidectomy in one series [9].

In some patients transoral placement of rigid trocars results in skin dimpling in the lower chin area is recognized by palpation which is caused by the rigid trocars. This alteration is almost always recognized by palpation and hardly ever visible and reversible during follow up.

A suggested set of postoperative exercises to hasten recovery is recommended to optimize functional outcome and to reduce scarring.

## Discussion

Transoral surgery vestibular approach to the central neck represents a feasible and safe solution for patients who attach great importance to avoid any cutaneous incision. Cosmetic results are excellent, thyroid surgery specific complication rates are equal to conventional thyroid surgery, and transoral specific new complications are rare. The vestibular access is more reasonable than other remote access procedures like axillary, inframammary or the pure retroauricular approach because of unfamiliar angle of vision and layers and difficulty to easily perform bilateral cervical surgery. However, further studies are necessary to evaluate the impact of vestibular trocar placement regarding short- or long-term restrictions of the mimetic muscles in the perioral, chin and submental region [28]. In this regard the authors did not observe permanent dysfunction of mimetic muscles so far.

TOETVA and TOEPVA have spread rapidly as an alternative to other extracervical remote access thyroidectomy techniques and to open thyroidectomy as well. With increasing operative use, surgeon experience and the potential to combine transoral surgery with other extracervical approaches, the gap regarding indications and outcomes between transoral surgery and the transcervical approach will likely continue to narrow, with both operative time and the incidence of specific complications diminishing. It is obvious that inclusion criteria for transoral thyroid surgery have broadened since the first 60 cases published in 2016, currently including also well differentiated carcinomas and thyroid glands >40 mL volume. However, learning curves must be considered and careful adoption of the technique is essential for successful implementation. Therefore, the authors emphasise cadaver training and proctoring by experienced surgeons within the framework of implementation of the transoral technique. In case that other remote access techniques in thyroid surgery are already implemented a combination of e.g. transaxillary, retoauricular, submandibular and transoral techniques might be an option, especially regarding removal of the specimen [27]. TOETVA is a solution for patients who wish to avoid any cutaneous incision and or have a history of hypertrophic scarring or keloids. Russel et al. estimate that about 55% of patients undergoing thyroid and parathyroid surgery may be eligible for transoral surgery taking the using the most widely accepted guidelines as a basis [8]. Some data suggest that most patients (Europe) are satisfied with their scar after thyroid surgery independent from the length of the incision and more than 80% would not have preferred an extracervical (transaxillary) procedure over the procedure they received. Contrariwise, as early as 2010 about 20% prevention of a visible scar in the neck would have been an option [29].

Currently more than 400 publications regarding transoral surgery are available, some focussing on anatomical or technical details and basics, further development and improvement of the technique, step by step implementation support, evaluation of potential risks or clinical outcomes in comparison to conventional, minimally invasive and/or other extracervical approaches, resulting in an increase of evidence and high level of transparency. However, further studies are necessary to demonstrate the long-term value of the approach.

Forthcoming technical development and improvement might facilitate remote access and transoral surgery, like single-port robotic assisted systems, although the development and implementation of transoral surgery may have high initial costs. Though, higher costs are accompanied by optimum cosmesis, less pain and improved quality of life representing determining factors for many patients.

## Supplementary Material

Supplementary Material
