# Supplementary material for: Transoral thyroid surgery vestibular approach
Source: Innov Surg Sci. 2022 Jun 17;7(3-4):107–13. doi: 10.1515/iss-2021-0033 (PMC9742272; doi:10.1515/iss-2021-0033)
Supplement: Supplementary file 1 — Supplementary Material [file j_iss-2021-0033_suppl.pdf]

Reviewer Assessment

E. Karakas, G. Klein & S. Schopf: Transoral Thyroid Surgery Vestibular Approach

Reviewers' Comments to Original Submission

Reviewer 1: Ozer Makay

Date received: 02-May-2021
Reviewer recommendation: **Return to author for minor modifications**
Reviewer overall scoring: **Medium**

Assessment Form scores: 5 = High/Yes; 3 = Medium/Adequate; 1 = Low

Is the subject area appropriate for the journal		4			
Does the title clearly reflect the paper's content?		4			
Does the abstract clearly reflect the paper's content			3		
Do the keywords clearly reflect the paper's content?		4			
Does the introduction present the problem clearly?			3		
Are the results/ conclusions justified?			3		
How comprehensive and up-to-date is the subject matter presented?		4			
How adequate is the data presentation?			3		
Are units and terminology used correctly?		4			
Is the number of cases adequate?		4			
Are the experimental methods/ clinical studies adequate?			3		
Is the length appropriate in relation to the content?				2	
Does the reader get new insights from the article?			3		
Please rate the practical significance.			3		
Please rate the accuracy of methods.			3		
Please rate the statistical evaluation and quality control.			3		
Please rate the appropriateness of the figures and tables.			3		
Please rate the appropriateness of the references.			3		
Please evaluate the writing style and use of language.		4			
Please judge the overall scientific quality of the manuscript.					
Are the methods used worthy of reproduction in greater deal?	No				
Would you be willing to review a revision of this manuscript?	Yes				

Comments to author: The present review entitled: "Transoral Thyroid Surgery Vestibular Approach", is an interesting, detailed, but moderate discussed paper. Authors are reporting the role of the transoral approach during thyroid surgery, trying to outline the topic from A to Z. The paper is interesting for the reader who wants to increase knowledge regarding this innovative technique. Technically, the procedure has been well described.

The focus of the current report seems to be in the target of the journal, but needs some revisions, due to considerable major limitations:

1) ABSTRACT:
• Methods: Please add that trocars, with gas outflow are warranted
• Results: Please add that experience in thyroid and endscopic surgery matters (This is advised to be mentioned also in the 'Qualifications & Requirements' section).

2) Recently there are more than 1000 cases (Anuwong's personal experience exceeds 2000).

3) TECHNIQUE:
• Maximum pressure should not exceed 6mmHg. Please correct
• Please describe which case receives the 5mm central trocar and in that particular case, how to extract the tissue.
• Submental use for tissue extraction is also an alternative. Authors are invited to add this to the text with referencing (Chen Y, Chomsky-Higgins K, Nwaogu I, Seib CD, Gosnell JE, Shen WT, Duh QY, Suh I. Hidden in Plain Sight: Transoral and Submental Thyroidectomy as a Compelling Alternative to "Scarless" Thyroidectomy. J Laparoendosc Adv Surg Tech A. 2018 Nov;28(11):1374-1377).
• Readers are possible applicants. So, do not encourage to leave the midline without closing at the end of the surgery. There is a risk that leaving the strap muscles non-sutured may result in adherence of the trachea to the skin.

4) Thyroiditis and Graves' disease should be mentioned as a relative contraindications.

5) Please add the followings to complications:
• Mucosal burn
• Perioral hematoma
• Mental nerve branch injury, esp. of the distal points, during introduction of the central port.
• Major vascular injury
• Tracheal perforation
• Skin dimpling in the lower chin area

6) Insertion and static & dynamic movements of trocars may result with complications. This has been stated in a paper by Celik et al. (Celik S, Makay O, Yoruk MD, Bayzit Kocer I, Ozdemir M, Kilic KD, Tomruk C, Bilge O, Uyanikgil Y, Dionigi G. A surgical and anatomo-histological study on Transoral Endoscopic Thyroidectomy Vestibular Approach (TOETVA). Surg Endosc. 2020 Mar;34(3):1088-1102).

7) DISCUSSION:
• Please discuss in more detail:
  - Eligibility
  - Scar perception of the patient requiring thyroid surgery
  - Evidence-based outcomes: level, controlled-trials, lack of randomized trials and the need for high level of evidence-based outcomes.

8) Please check Reference number 8.

9) Figure 8 is not clear and informative. Withdrawal is recommended.

10) Figure 10 is not clear. Please use another one , with better resolution.

Reviewer 2: Christian Scheuba

Date received: 01-Jul-2021
Reviewer recommendation: **Accept in present form**
Reviewer overall scoring: **High**

Assessment Form scores: 5 = High/Yes; 3 = Medium/Adequate; 1 = Low

Is the subject area appropriate for the journal	5				
Does the title clearly reflect the paper's content?	5				
Does the abstract clearly reflect the paper's content	5				
Do the keywords clearly reflect the paper's content?	5				
Does the introduction present the problem clearly?	5				
Are the results/ conclusions justified?		4			
How comprehensive and up-to-date is the subject matter presented?		4			
How adequate is the data presentation?		4			
Are units and terminology used correctly?	5				
Is the number of cases adequate?	5				
Are the experimental methods/ clinical studies adequate?		4			
Is the length appropriate in relation to the content?		4			
Does the reader get new insights from the article?	5				
Please rate the practical significance.		4			
Please rate the accuracy of methods.		4			
Please rate the statistical evaluation and quality control.		4			
Please rate the appropriateness of the figures and tables.			3		
Please rate the appropriateness of the references.	5				
Please evaluate the writing style and use of language.		4			
Please judge the overall scientific quality of the manuscript.		4			
Are the methods used worthy of reproduction in greater deal?	No				
Would you be willing to review a revision of this manuscript?	n.a.				

Comments to author: nice work, low number of complications!

Recommendation by the Guest-Editor-on the Original Submission

Revise with Minor Modifications

Authors' Response to Reviewer Comments

Date received: 13-Jul-2021

Response to reviewer 1

Reviewer: 1

Comments to the Author
The present review entitled: "Transoral Thyroid Surgery Vestibular Approach", is an interesting, detailed, but moderate discussed paper. Authors are reporting the role of the transoral approach during thyroid surgery, trying to outline the topic from A to Z. The paper is interesting for the reader who wants to increase knowledge regarding this innovative technique. Technically, the procedure has been well described.

The focus of the current report seems to be in the target of the journal, but needs some revisions, due to considerable major limitations:

1) ABSTRACT:
• Methods: Please add that trocars, with gas outflow are warranted
Answer: The authors added: "An additional device to optimize gas outflow for optimum view might be helpful."

• Results: Please add that experience in thyroid and endscopic surgery matters (This is advised to be mentioned also in the 'Qualifications & Requirements' section).
Answer: The authors added: "Experience in thyroid and endoscopic surgery is required to achieve excellent results with low complication rates." Not in the "Results" but in the "Conclusion" part and amended the "Qualifications & requirement`s" section (PAGE 5 LINE 32) as follows: "Surgeon skills and qualification including experience in endoscopic surgery are of utmost importance for procedure safety. Proficiency in the standard procedures of central neck surgery, including thyroidectomy, parathyroidectomy, and central neck dissection in case of necessary conversion to open surgery is prerequisite [15,16]. An adequate volume is required to overcome the learning curve."

2) Recently there are more than 1000 cases (Anuwong's personal experience exceeds 2000).
Answer: Maybe true but not published yet. The authors added this in the "Introduction" (PAGE 5 LINE 13) part as follows: "Currently Anuwongs` personal experience exceeds 2000 cases (unpublished data) and..."

3) TECHNIQUE:
• Maximum pressure should not exceed 6mmHg. Please correct
Answer: The authors experience is that gas pressure can be raised up to 8mmHg. However, the section was changed (PAGE 6 LINE 53) to " High flow CO2 gas insufflation at a pressure of 6 mmHg is recommended."

• Please describe which case receives the 5mm central trocar and in that particular case, how to extract the tissue.
Answer: The authors added (PAGE 6 LINE 24): "A 5mm instead of a 10-12mm trocar combined with a 5mm 30o camera can be used in the midline. Use of the midline 5mm trocar may facilitate initial trocar placement. It is minimal invasive and space saving during surgery and not confined to particular cases. To extract taller specimen, tissue in the vestibular and chin area can be stretched at the end of surgery if necessary. Otherwise, additional extracervical or a submental approach can be used to extract huge thyroid specimen. 3mm trocars and instruments are available and applicable instead of the lateral 5mm incisions to reduce invasiveness."

• Submental use for tissue extraction is also an alternative. Authors are invited to add this to the text with referencing (Chen Y, Chomsky-Higgins K, Nwaogu I, Seib CD, Gosnell JE, Shen WT, Duh QY, Suh I. Hidden in Plain Sight: Transoral and Submental Thyroidectomy as a Compelling Alternative to "Scarless" Thyroidectomy. J Laparoendosc Adv Surg Tech A. 2018 Nov;28(11):1374-1377).
Answer: The authors added this reference, although submental skin incision is not really extracervical.

• Readers are possible applicants. So, do not encourage to leave the midline without closing at the end of the surgery. There is a risk that leaving the strap muscles non-sutured may result in adherence of the trachea to the skin.

Answer: The authors disagree. Some endocrine surgeons also do not close the midline but only adapt the platysm at the end of open or MIVA thyroid surgery. In our patients we did not recognize long term problems. Adherence will also occur in patients after open surgery and closure of the midline and the platysm.
As a compromise the authors suggest (PAGE 7 LINE 36): “Strap muscles can be adapted by transoral running suture at the end of the operation while the skin-platysm conjunction stays unaffected”.

4) Thyroiditis and Graves’ disease should be mentioned as a relative contraindications.

Answer: The authors disagree. Both diseases may aggravate the procedure but are not contraindications. The authors added (PAGE 8 LINE 30): “Like in conventional surgery thyroiditis may aggravate preparation and mobilization of the thyroid and transoral surgery should be performed by well experienced surgeons.”

5) Please add the followings to complications:
• Mucosal burn
• Perioral hematoma
• Mental nerve branch injury, esp. of the distal points, during introduction of the central port.
• Major vascular injury
• Tracheal perforation
• Skin dimpling in the lower chin area

Answer: The authors added the following regarding “Skin dimpling” (PAGE 10 LINE 47): “In some patients the transoral placement of rigid trocars results in skin dimpling in the lower chin area. This procedure specific alteration is almost always recognized by palpation and hardly ever visible. It is reversible during follow up.”
Mucosal burn is completely unknown to the authors? As well as perioral hematoma. The authors mentioned hematoma in the chin region and the neck but have never recognized hematoma around the mouth or around the upper lip.
The authors already mentioned mental nerve injury which is mainly caused by lateral trocar placement. Discussing the approaches with Head and Neck surgeons almost every surgeon emphasises that a transverse midline incision does not cause relevant damage to the mental nerve in ENT procedures and the length of the incision often exceeds 3cm in ENT surgery.
Major vascular injury and tracheal perforation are not transoral procedure specific and can also occur in conventional, transaxillary, ABBA or MIVAT surgery.
The authors added (PAGE 9 LINE 52): “In addition to common minor and major complications in thyroid surgery, like bleeding, vascular injury, RLN palsy, hypoparathyroidism or tracheal injury some procedure specific complications may occur in transoral surgery.” And ((PAGE 10 LINE 5) “Transient superficial hematoma of the skin region and neck may occur in some patients.”

6) Insertion and static & dynamic movements of trocars may result with complications. This has been stated in a paper by Celik et al. (Celik S, Makay O, Yoruk MD, Bayzit Kocer I, Ozdemir M, Kilic KD, Tomruk C, Bilge O, Uyanikgil Y, Dionigi G. A surgical and anatomo-histological study on Transoral Endoscopic Thyroidectomy Vestibular Approach (TOETVA). Surg Endosc. 2020 Mar;34(3):1088-1102).
The authors added the paper in the discussion part as follows (PAGE 11 LINE 21): “However, further studies are necessary to evaluate the impact of vestibular trocar placement regarding short- or long-term restrictions of the mimetic muscles in the perioral, chin and submental region [30]. In this regard the authors did not observe permanent dysfunction of mimetic muscles so far.”

7) DISCUSSION:
• Please discuss in more detail:

Eligibility and scar perception.
The authors added (PAGE 12 LINE 1): “TOETVA is a solution for patients who wish to avoid any cutaneous incision and or have a history of hypertrophic scarring or keloids. Russel et al. estimate that about 55% of patients undergoing thyroid and parathyroid surgery may be eligible for transoral surgery taking the using the most widely accepted guidelines as a basis [8]. Some data suggest that most patients (Europe) are satisfied with their scar after thyroid surgery independent from the length

of the incision and more than 80% would not have preferred an extracervical (transaxillary) procedure over the procedure they received. Contrariwise, as early as 2010 about 20% prevention of a visible scar in the neck would have been an option [31].

Evidence-based outcomes: level, controlled-trials, lack of randomized trials and the need for high level of evidence-based outcomes.

The authors added (PAGE 12 LINE 1): Currently more than 400 publications regarding transoral surgery are available, some focussing on anatomical or technical details and basics, further development and improvement of the technique, step by step implementation support, evaluation of potential risks or clinical outcomes in comparison to conventional, minimally invasive and/or other extracervical approaches, resulting in an increase of evidence and high level of transparency. However, further studies are necessary to demonstrate the long-term value of the approach.

8) Please check Reference number 8.
Reference checked and added.

9) Figure 8 is not clear and informative. Withdrawal is recommended.
Figure 8 has been removed.

10) Figure 10 is not clear. Please use another one, with better resolution.
Figure 10 has been changed.

Comments by the Editor-in-Chief to Revised Submission – Final Decision

All reviewer comments were addressed by the authors. I accept the revised manuscript for publication in our journal.
